# Multi-omics analysis identifies *FoxO1* as a regulator of macrophage function through metabolic reprogramming

**DOI:** 10.1038/s41419-020-02982-0

**Published:** 2020-09-24

**Authors:** Kai Yan, Tian-Tian Da, Zhen-Hua Bian, Yi He, Meng-Chu Liu, Qing-Zhi Liu, Jie Long, Liang Li, Cai-Yue Gao, Shu-Han Yang, Zhi-Bin Zhao, Zhe-Xiong Lian

**Affiliations:** 1 Liver Immunology Laboratory, School of Life Sciences, University of Science and Technology of China, Hefei, 230022 Anhui China; 2 Chronic Disease Laboratory, Institutes for Life Sciences and School of Medicine, South China University of Technology, Guangzhou, 510006 Guangzhou China; 3 Department of Rheumatology and Immunology, The Third Affiliated Hospital of Southern Medical University, Guangzhou, 510630 Guangdong China

**Keywords:** Cell biology, Immunology

## Abstract

Macrophages are plastic cells that can switch among different states according to bioenergetic or biosynthetic requirements. Our previous work demonstrated that the transcription factor Forkhead Box Protein 1 (*FoxO1*) plays a pivotal role in regulating the function of macrophages, but the underlying mechanisms are still unclear. Here we identify *FoxO1* as a regulator of macrophage function through metabolic reprogramming. Transcriptomic and proteomic analyses showed that the deficiency of *FoxO1* results in an alternatively activated (M2) phenotype of macrophages, with lower expression of inflammatory response- and migration-associated genes. Using the high content screening and analysis technology, we found that deletion of *FoxO1* in macrophages slows their migration rate and impairs their function to limit tumor cell growth in vitro. Next, we demonstrated that glycolysis is inhibited in *FoxO1*-deficient macrophages, which leads to the observed functional changes and the reduced tumor suppression capability. This prospective study shows that *FoxO1* serves as a bridge between metabolism and macrophage function.

## Introduction

Macrophages form a heterogeneous population of antigen-presenting and tissue-resident phagocytic cells. They develop from the terminal differentiation of circulating monocytes or from E8.0 yolk sac^1–3^. Macrophages are central effector cells of the host defense mechanism; they phagocytize and digest impurities, dead cells, and pathogens, and they also play important roles in the orchestration of innate and adaptive immune responses^4,5^. Resting state (M0) macrophages, derived from the bone marrow, usually function as precursors of polarized macrophages. Upon stimulation with lipopolysaccharide (LPS) and Th1 pro-inflammatory cytokines, M0 cells can be polarized to classically activated (type 1 or M1) pro-inflammatory macrophages and interleukin *(IL)-4*/*IL-13*, or *IL-10*/transforming growth factor-β stimulation results in alternatively activated (type 2 or M2) anti-inflammatory macrophages^6–8^. M1 macrophages initiate and sustain the inflammatory responses, by secreting cytokines such as tumor necrosis factor-α (*TNF-α*), *IL-6*, and interferon-γ (*IFN-γ*), and recruiting other immune cells to the inflamed tissue^9,10^. Contrary to M1 macrophages, M2 macrophages exhibit an anti-inflammation phenotype; they promote the resolution of inflammation, phagocytize apoptotic cells, and release Th2-inflammatory mediators such as *IL-4*^8,11^. In tumor, the macrophage polarization become more complex. Tumor-associated macrophages (TAMs) adopt activation signals over a wide time range, to express M1-associated genes and more M2-associated genes^12–15^. Tumor cells harness metabolic byproducts to keep immune cells in their favor. The secretion of large amounts of lactate favors the polarization to an immunosuppressive phenotype^16,17^. It is known that macrophages display diverse metabolic states, especially macrophages polarized in vitro. In M1 macrophages, an increased glycolysis rate is closely associated with an inflammatory phenotype, including the secretion of pro-inflammatory cytokines and opsonin-mediated phagocytosis^10,18,19^. In contrast, M2 macrophages employ oxidative phosphorylation as their main energy source^20–22^. TAMs show high glycolytic activity and high oxidative phosphorylation, which means that TAMs present M1 and M2 characteristics with heterogenous subpopulations^17^. It is known for dramatic switches in metabolic signature of macrophages governing its phenotype and function^16,23–26^. However, little is known about the transcriptional mechanisms underlying metabolic reprogramming in macrophages.

The transcription factor Forkhead Box Protein 1 (*FoxO1*) is a member of the Forkhead Box family and plays significant roles in a series of cellular processes, including inflammation, metabolism, and signal transduction^27–30^. In tumor cells, *FoxO1* is generally considered as a tumor suppressor and upregulation of *FoxO1* is beneficial in several types of cancer^31–33^. In macrophages, *FoxO1* seems to have various functions. *FoxO1* dampens macrophage inflammation without promoting apoptosis in the context of endoplasmic reticulum stress^4^ and it also promotes goblet cell hyperplasia in asthma by regulating M2-like macrophage activation^34^. Besides *FoxO1* also interacts with the Toll-like receptor 4-mediated inflammatory response^35^ and regulates *IL-10* secretion during classic activation in hyperglycemia^36^. In previous studies, we found that *FoxO1* regulates macrophage polarization in infection and promotes the anti-tumor function of TAMs by upregulating major histocompatibility complex II (*MHC-II*) expression^37,38^. In this study, we focused on the transcriptional mechanism of macrophage metabolic reprogramming.

## Results

### *FoxO1*^mKO^ macrophages are skewed toward an M2 phenotype in physiological state

**Fig. 1:**
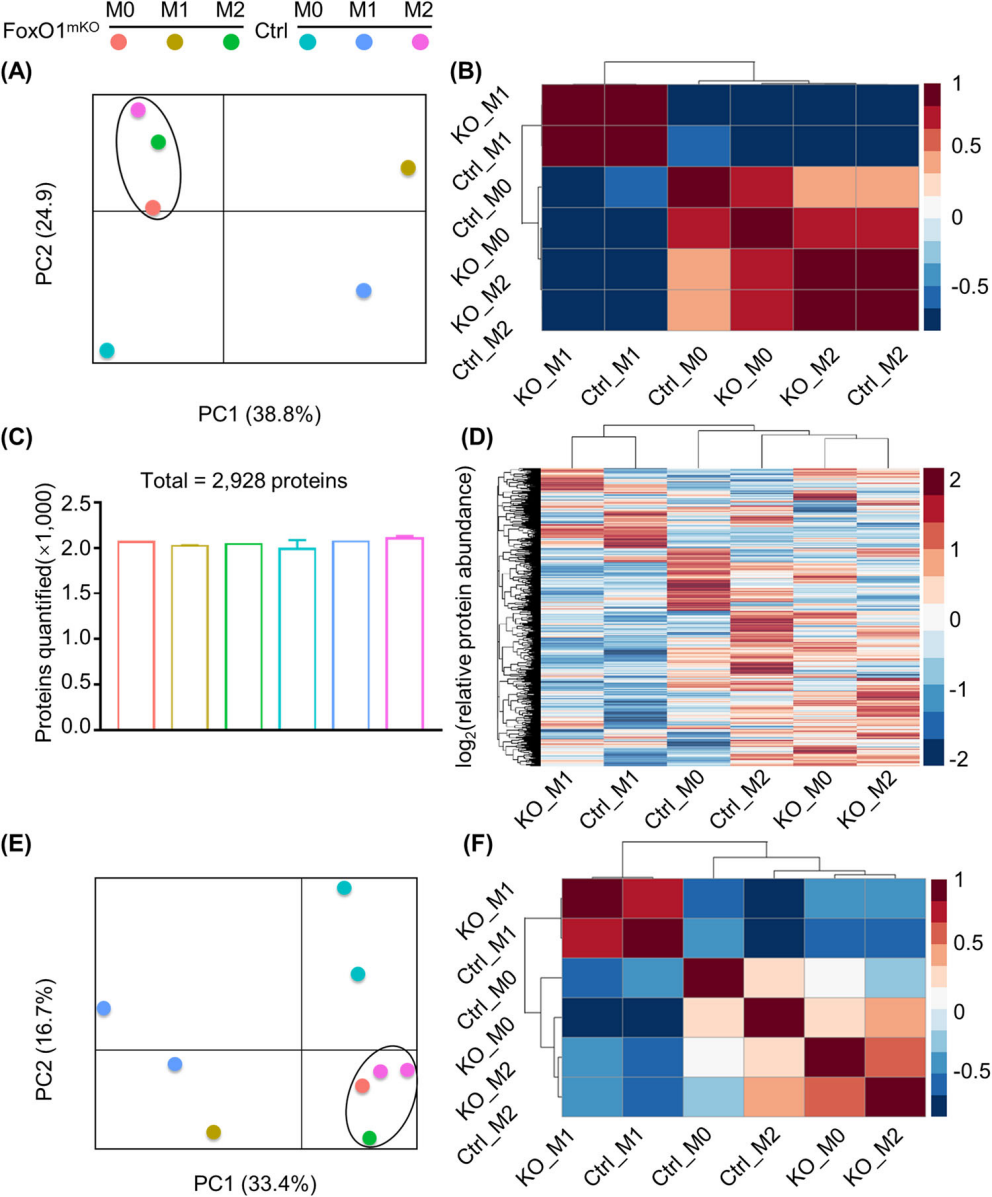
Transcriptome and proteome analyses imply propinquity of the *FoxO1*^mKO^ macrophage phenotype to that of alternatively activated (M2) macrophages. The transcriptome and proteome of M0, M1, and M2 macrophages from *FoxO1*^mKO^ and littermate control mouse bone marrow-derived macrophages (BMDMs) were analyzed by RNA sequencing and proteomics analysis. **a** A 2D principal component analysis (PCA) plot of the transcriptome data that characterizes the trends exhibited by the expression profile of 21,460 genes of *FoxO1*^mKO^ M0 (orange), *FoxO1*^mKO^ M1 (gray), *FoxO1*^mKO^ M2 (green), Ctrl M0 (cyan), Ctrl M1 (blue), and Ctrl M2 (pink) BMDMs. Every transcriptome sequencing sample is a mixture of two *FoxO1*^mKO^ mice or two littermates. **b** Pearson’s correlation computation of transcriptome data in all samples. Colors indicate the coefficient values between classes of cells. **c** Numbers of unique proteins quantified by Sequest-HT in BMDMs; the total number of identified proteins was 2928. The bar color is the same as the color of Fig. 1a’s dot. **d** Consensus-clustering analysis of proteomic profiling identifies six BMDM proteomic subtypes (*n* = 9). The heat map depicts the relative abundance of signature proteins (log_2_-transformed). The PCA plot (**e**) and Pearson’s correlation analysis (**f**) of the proteomics data by the total 2400 efficient protein expression profiles of BMDM cells. Figure 1a, e share the same dot color.

*FoxO1* is a diversified transcriptional factor but little is known about its role in macrophage metabolism and function. First, we cultured bone marrow-derived macrophages (BMDMs) from *FoxO1* myeloid-specific knockout (KO) mice (*FoxO1*^mKO^) and their littermate controls (*FoxO1*^fl/fl^). We found that *foxo1* deficiency did not affect macrophage development in vivo (Supplementary Fig. S2). Next, RNA and protein were extracted separately for further transcriptome and proteome analyses. A total of 21,460 efficient genes in all samples (*FoxO1*^mKO^ M0, *FoxO1*^mKO^ M1, *FoxO1*^mKO^ M2, *FoxO1*^fl/fl^ M0, *FoxO1*^fl/fl^ M1, and *FoxO1*^fl/fl^ M2 BMDMs) were analyzed (Gene Expression Omnibus (GEO): GSE97260). Principal component analysis (PCA) and Pearson’s correlation results of transcriptome data indicate that the physiological state of *FoxO1*^mKO^ macrophages is more similar to that of control M2 cells than control M0 cells (Fig. 1a, b). Our proteomics data are consistent with the transcriptome results. Here we examined the quantified proteins of each samples, which identified ~2000 proteins, and there was no difference in protein abundance between *FoxO1*^mKO^ and *FoxO1*^fl/fl^ macrophages (Fig. 1c). PCA and Pearson’s correlation results of proteomics data also showed that the physiological state of *FoxO1*^mKO^ macrophages resembles that of a M2 phenotype (Fig. 1d–f). These results suggest that *FoxO1* deficiency results in an M2 phenotype of macrophages.

### *FoxO1* regulates the polarization and function of macrophages

**Fig. 2:**
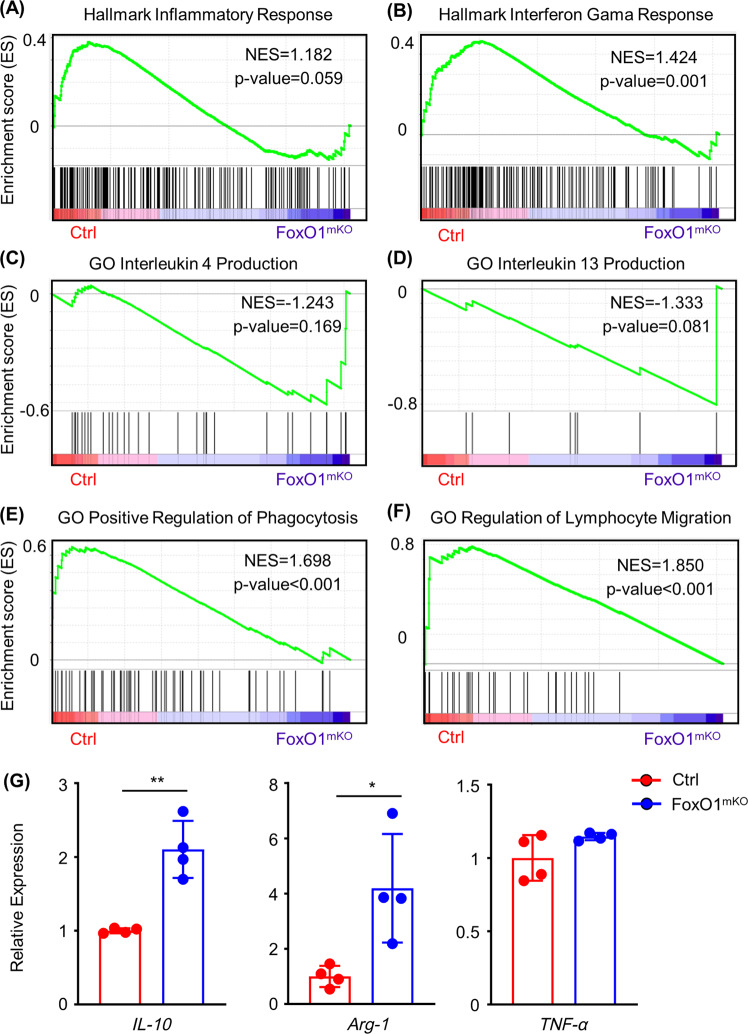
Multi-profiling implies *FoxO1* deficiency results in resemblance of the phenotype and function of macrophages to those of alternatively activated (M2) macrophages. The transcriptome profiles of macrophages from *FoxO1* conditional knockout mice and littermates were analyzed by GSEA and confirmed with real-time-PCR. The list of differentially expressed genes (upregulated or downregulated) in Control and *FoxO1*-knockout macrophages was obtained from the MSigDB database. Two *FoxO1*^mKO^ mice or two littermates were mixed for transcriptomics. **a**–**f** GSEA plots of (**a**, **b**) pro-inflammatory gene sets, (**c**, **d**) anti-inflammatory cytokine production gene sets, (**e**) macrophage phagocytosis-related gene sets, and (**f**) macrophage migration-related gene sets. NES, normalized enrichment score. **g** Bar graphs showing the relative expression of *IL-10*, *Arg-1*, and *TNF-α* in control and *FoxO1*^mKO^ mice bone marrow-derived macrophages. Red bar represents control macrophages and the blue bar represents *FoxO1*-deficient macrophages. Four mice were used for the induction of bone marrow-derived macrophages in each experiment. *P < 0.05; **P < 0.01; ***P < 0.001.

To further confirm the role of *FoxO1* in macrophages, we used multi-profiling to explore the phenotype and function of *FoxO1*-deficient macrophages. First, we compared the typical gene expression sets in physiological states of *FoxO1*-deficient macrophages with those of control macrophages. As for pro-inflammatory genes, the “Hallmark Inflammatory Response” (normalized enrichment score (NES) = 1.182, *P* = 0.059; Fig. 2a), “Hallmark Interferon Gamma Response” (NES = 1.424, *P* = 0.001; Fig. 2b), and “Hallmark Interferon Alpha Response” (Supplementary Fig. S1A) gene sets showed significant downregulation in *FoxO1*-deficient macrophages compared with control macrophages. Regarding anti-inflammatory gene sets, “GO *IL4* Production” (NES = − 1.243, *P* = 0.169; Fig. 2c) and “GO *IL13* Production” (NES = − 1.333, *P* = 0.081; Fig. 2d) showed an enriched trend in *FoxO1*-deficient macrophages. Furthermore, gene set enrichment analysis (GSEA) plot of the “Positive Regulation of Macrophage Phagocytosis” (NES = 1.698, *P* < 0.001; Fig. 2e) and “GO Macrophage Activation Involved in Immune Response” (NES = 1.726, *P* = 0.003; Supplementary Fig. S1B) gene sets showed significant downregulation in *FoxO1*-deficient macrophages. In addition, the macrophage chemotaxis- and migration-related gene sets “GO Regulation of Macrophage Chemotaxis” (NES = 1.497, *P* = 0.032; Supplementary Fig. S1C) and “GO Regulation of Lymphocyte Migration” (NES = 1.850, *P* < 0.001; Fig. 2f) showed significant downregulation in *FoxO1*-deficient macrophages. Finally, quantitative PCR and flow cytometry were used to confirm the mRNA and protein expression levels of M1/M2-associated markers in sh*FoxO1* lentivirus-infected macrophages and *FoxO1*^mKO^ macrophages. As shown in Fig. 2g, *IL-10* and *Arg-1* (M2 markers) expression significantly increased in *FoxO1*-deficient macrophages, but there was no difference on *TNF-α* (an M1 marker). The flow cytometry results showed that *CD206* (an M2 marker) was upregulated but *MHC-II* (an M1 marker) was downregulated in *FoxO1*^mKO^ macrophages (Supplementary Fig. S1E). Besides, the expression levels of *IL-4*, a cytokine enriched in M2 macrophages, were significantly increased in *FoxO1*-deficient macrophages compared with normal macrophages, but no differences in the expression of *IL-6* (an M1 cytokine) were observed (Supplementary Fig. S1D).

### *FoxO1*-deficient macrophages exhibit an M2-like phenotype

**Fig. 3:**
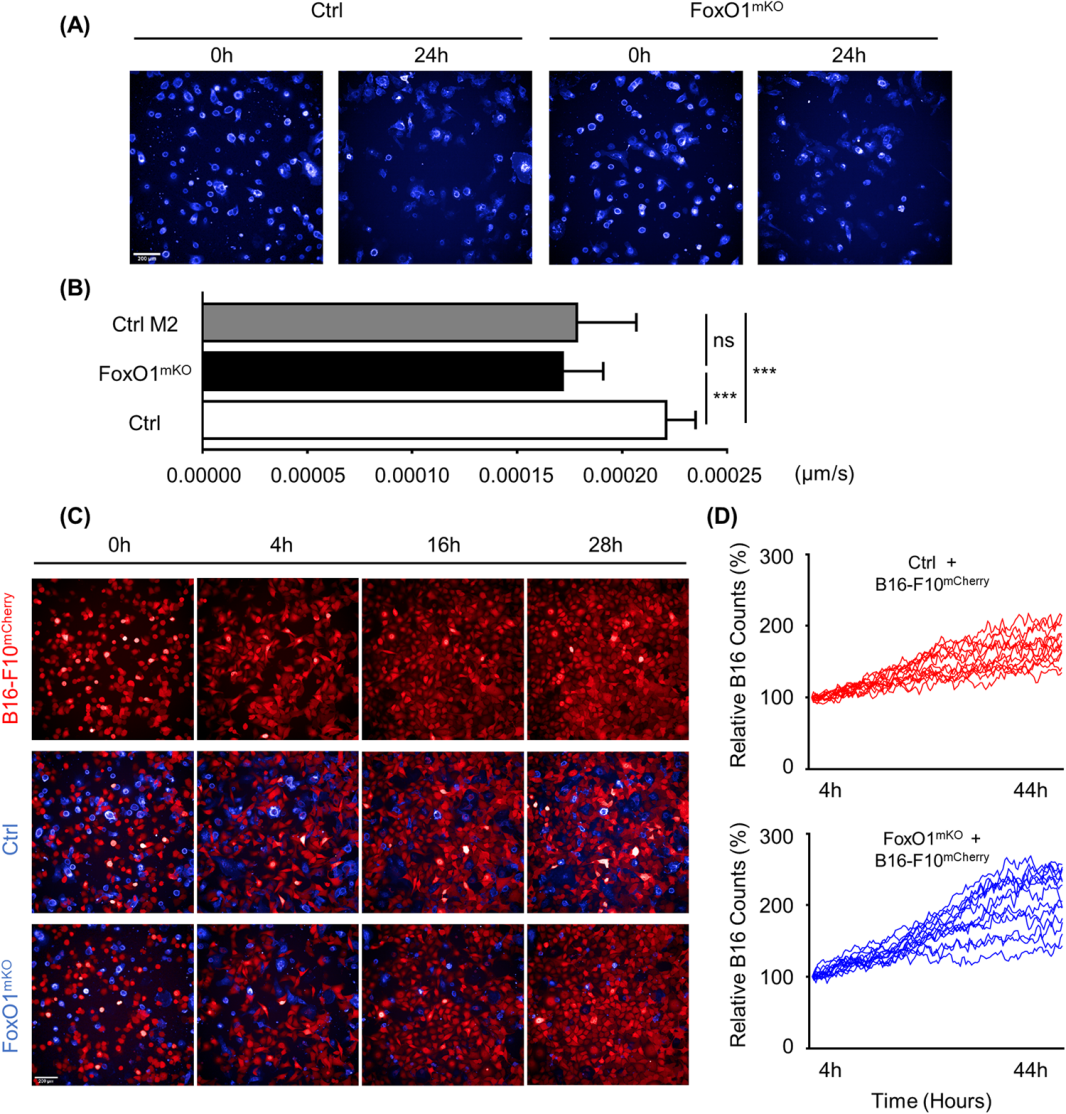
In vitro system shows that *FoxO1*^mKO^ macrophages have an M2-like phenotype. *FoxO1*-knockout and control macrophages were cultured and analyzed in 96-well plates with the high content screening and analysis technology (HCS). **a** Images showing (right) APC-F4/80 labeled *FoxO1*^mKO^ and (left) control macrophages after 24 h. The magnification is ×200 and the scanning frequency was three times an hour. **b** Bar graph presenting the mean moving speed (μm/s) of cells per vision (*n* ≥ 100). The black bar represents *FoxO1*^mKO^ macrophages. The white bar represents control macrophages. The gray bar represents M2 macrophages. **c** HCS images showing the co-culture results of (lower) *FoxO1*^mKO^ macrophages and B16-F10^mCherry^ cells, (middle) macrophages and B16-F10^mCherry^ cells, and (upper) B16-F10^mCherry^ cells only, at 0, 4, 16, and 28 h. Blue cells are macrophages and red cells are B16-F10 cells. **d** Dynamic graph of B16 cell counts relative to the 4 h time point (*n* ≥ 9). Cells were counted every 20 min. Blue lines represent relative counts of *FoxO1*^mKO^ macrophages co-cultured with B16 cells. Red lines represent relative counts of control macrophages co-cultured with B16 cells. At 28 h, *P* = 0.0299 and at 44 h, *P* = 0.0095. Scale bar, 200 μm. *P < 0.05; **P < 0.01; ***P < 0.001.

Our transcriptome and proteome analyses showed that *FoxO1*-deficient macrophages resemble M2 macrophages in phenotype and function. Next, we further explored the role of *FoxO1* in macrophage migration and phagocytosis. First, we used the high content screening and analysis technology (HCS) system to calculate the moving speeds of *FoxO1*^mKO^ and *FoxO1*^fl/fl^ macrophages. We found that the lack of *FoxO1* in macrophages leads to decreased moving speeds (Fig. 3a, b). In addition, when co-cultured with B16-F10^mCherry^ cells, the moving speed of *FoxO1*^mKO^ macrophages increased, but they remained slower than normal macrophages (Supplementary Fig. S3A). Moreover, we observed the phagocytosis function of macrophages by HCS co-culturing at different time points and compared the relative B16-F10^mCherry^ counts (Fig. 3c, d, Supplementary Fig. S3D, and Supplementary Video S1A, B). As shown in Supplementary Fig. S3D, the relative B16 counts gradually decreased from M2, M0, to M1 in co-culture system. The relative proportion of B16 counts significantly enhanced when co-cultured with *FoxO1*-deficient macrophages compared with control macrophages (*p* < 0.05) (Fig. 3c, d and Supplementary Video S1A, B). The phagocytosis process was also directly visualized by merging HCS and immunofluorescence videos (Supplementary Fig. S3B, C and Supplementary Videos S2 and S3A, B). Consistent with the lower moving speed, the anti-tumor function (including phagocytosis) of *FoxO1*^mKO^ macrophages was impaired compared to that of control macrophages. These data indicate that *FoxO1* not only regulates the differentiation of macrophages but also promotes moving and anti-tumor effect including phagocytosis, especially when co-cultured with cancer cells.

### Glycolytic activity is downregulated in *FoxO1*-deficient macrophages

**Fig. 4:**
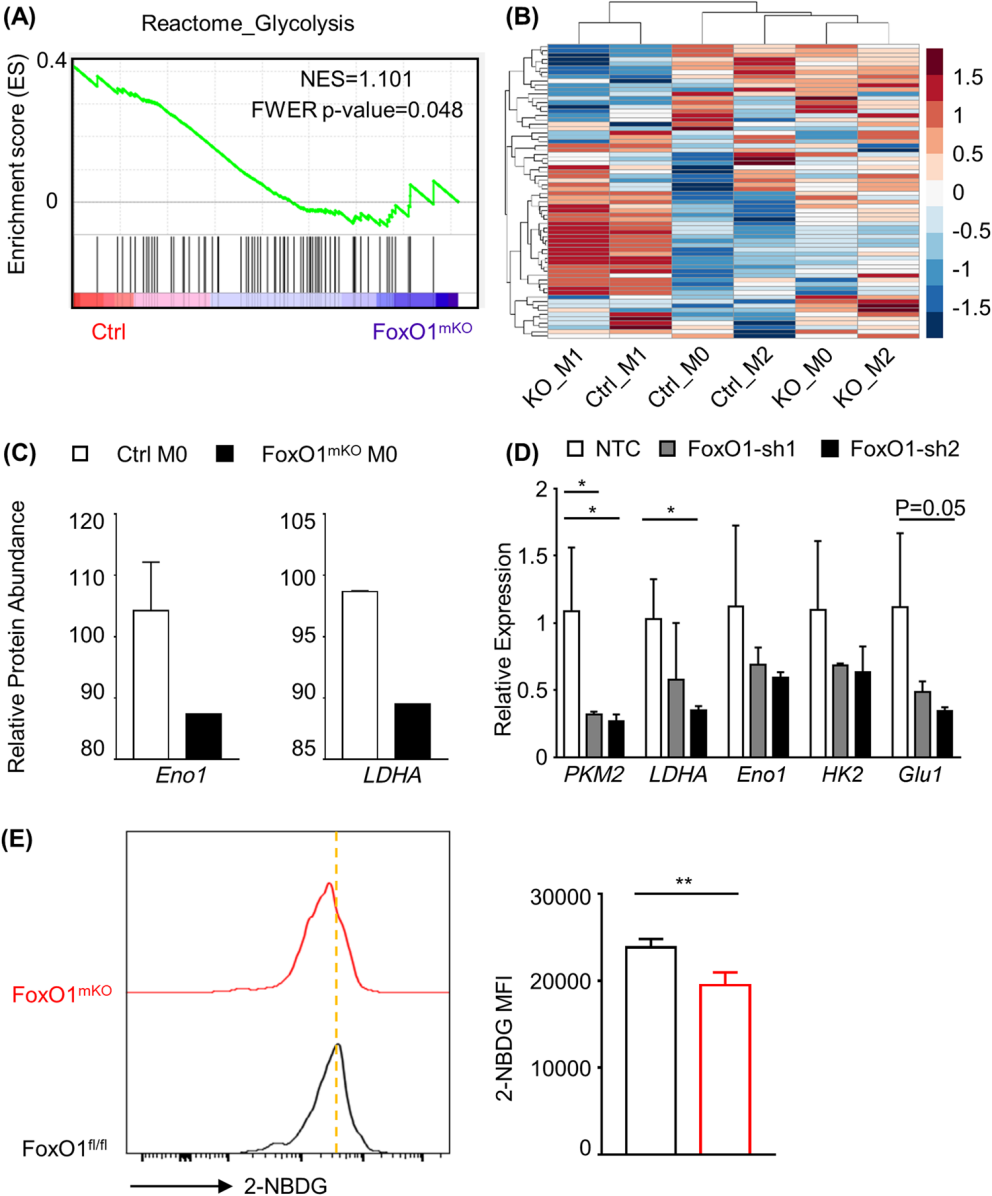
*FoxO1*-deficient macrophages exhibit reduced glycolytic activity. **a** Gene set enrichment analysis (GSEA) of the “Reactome Glycolysis” gene set in *FoxO1* conditional knockout macrophages vs. control macrophages was performed on the RNA-seq data set. **b** Heat map depicting color-coded expression levels of genes from the “Reactome Glycolysis” gene set in *FoxO1*^mKO^ and control M0, M1, and M2 BMDMs, as obtained by GSEA. **c** Relative expression levels of key glycolysis genes *LDHA* and *Eno1* from the proteome data set. The black bar represents *FoxO1*^mKO^ macrophages. The white bar indicates control macrophages. **d** Expression levels of glycolysis-related genes were determined by quantitative real-time PCR (*n* ≥ 3). **e** 2-NBDG fluorescence intensity in *FoxO1*^mKO^ macrophages (red) and control macrophages (black). MFI, mean fluorescence intensity (*n* ≥ 3). *P < 0.05; **P < 0.01; ***P < 0.001.

It is well known that metabolic homeostasis defines functions, especially glucose metabolism, where the metabolite concentrations (e.g., succinate) directly alter the activity and function of signaling pathways. Interestingly, our GSEA showed that the profile of glycolysis-associated genes in *FoxO1*^mKO^ macrophages resembled that of M2 macrophages more than it resembled that of *FoxO1*^fl/fl^ macrophages (Fig. 4a). Our random clustered heat map of glycolysis-associated genes in *FoxO1*^mKO^ macrophages and *FoxO1*^fl/fl^ macrophages shows that glycolytic activity in *FoxO1*-deficient macrophages is similar to that in M2 macrophages (Fig. 4b). The genes associated with Reactome glycolysis reduced in *FoxO1*^mKO^ macrophages compared with those of *FoxO1*^fl/fl^ macrophages in physiological state according to proteomics data (Fig. 4c). We also performed short hairpin RNA (shRNA) knockdown of *FoxO1* to further confirm the role of *FoxO1* in regulating glycolytic activity. As shown in Fig. 4d, knockdown of *FoxO1* in macrophages with shRNA results in reduced expression of the key glycolytic molecules glucose transporter 1 (*Glut1*), lactate dehydrogenase A (*LDHA*), enolase 1 (*Eno1*), pyruvate kinase isozymes M2 (*PKM2*), and hexokinase 2 (*HK2*). *FoxO1*^fl/fl^ macrophages infected with adeno-cre virus further validated the downregulation of *Glut1*, *Eno1*, *PKM2*, and *HK2* genes along with FoxO1 deficiency (Supplementary Fig. S4A, B). Besides, the glycolytic gene expression pattern in *FoxO1*-deficient macrophages showed a similar low-expression manner with M2 macrophages, but different from the high-glycolysis M1 macrophages (Supplementary Fig. S4A, B). We also confirmed the reduced glucose uptake level by flow cytometry in *FoxO1*-KO BMDMs (in vitro) (Fig. 4e) and peritoneal cavity (PC) macrophages (ex vivo) (Supplementary Fig. S4C). Besides, both *FoxO1* conditional KO BMDMs and PC macrophages showed a reduced lactate production trend compared with control macrophages (Supplementary Fig. S4D, E). Collectively, these results demonstrate that glycolytic activity in macrophages is downregulated upon *FoxO1* deficiency, indicating that *FoxO1* is a regulator of macrophage function through metabolic reprogramming.

### *FoxO1* regulates glucose metabolism of macrophages

**Fig. 5:**
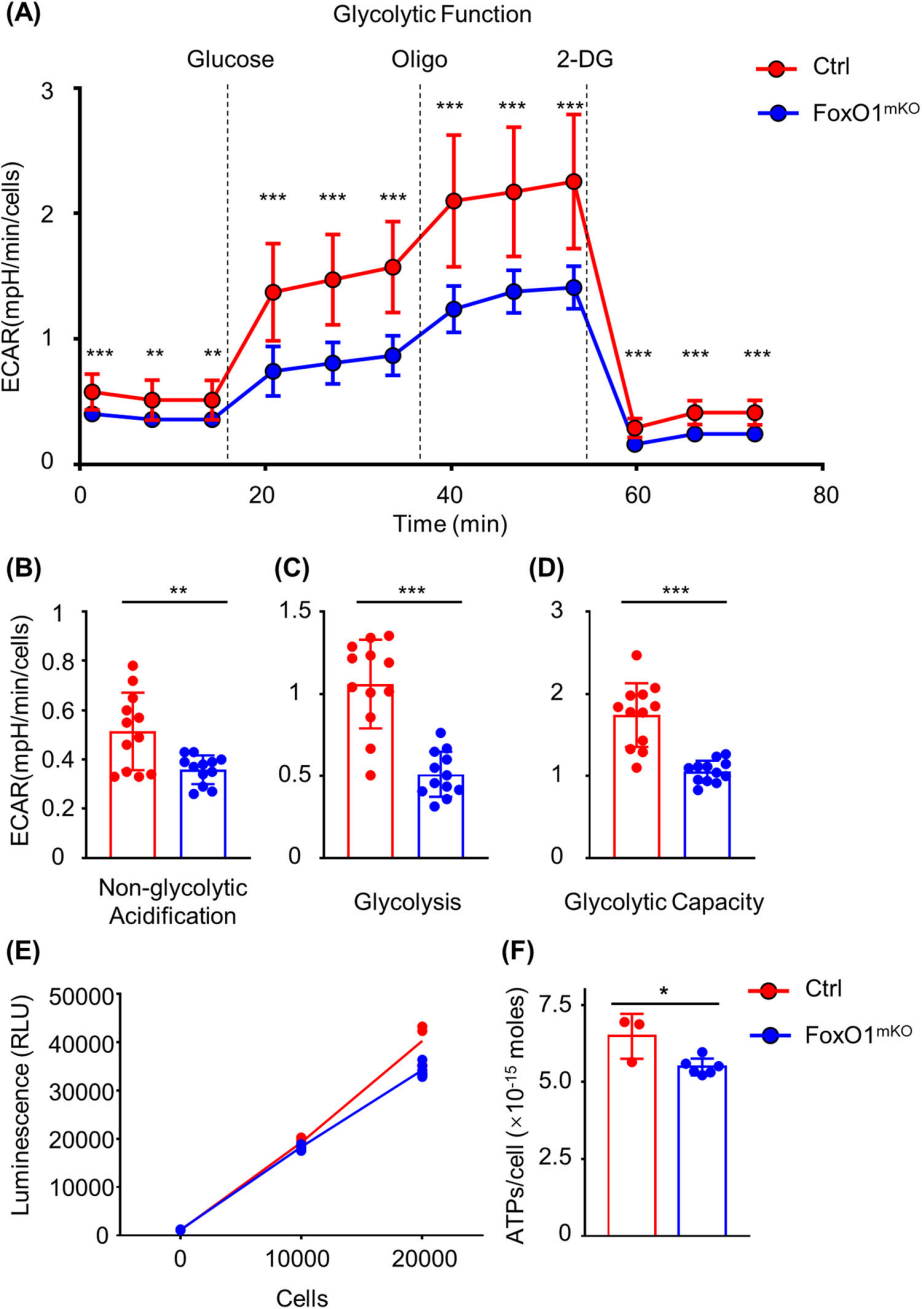
Glycolysis stress test showed a decrease in glycolytic activity in *FoxO1* conditional knockout macrophages. **a** Kinetic ECAR response of *FoxO1*^mKO^ (blue line) and control (red line) macrophages to glucose (10 mM), oligomycin (1.0 μM), and 2-DG (50 mM) (*n* ≥ 3). Macrophages were plated at 80,000 cells per well in XFe96 cell culture plates 1 day prior to the assays. **b–d** Statistical analysis of (**b**) non-glycolytic acidification, (**c**) glycolysis, and (**d**) glycolytic capacity in *FoxO1* conditional knockout macrophages vs. control macrophages. **e** Luminescent signal of *FoxO1*^mKO^ (blue), control (red) macrophages and cell counts. **f** Statistical analysis of ATP per cells of *FoxO1*^mKO^ (blue), control (red) macrophages (*n* ≥ 3). *P < 0.05; **P < 0.01; ***P < 0.001.

**Fig. 6:**
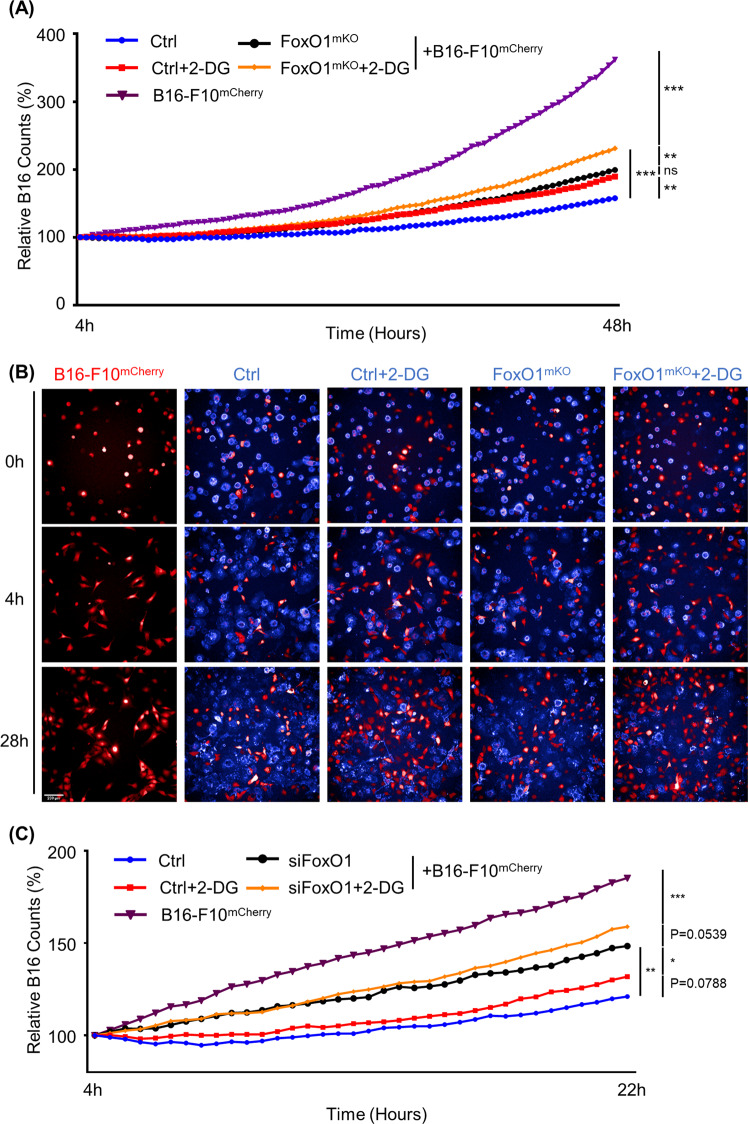
Glycolysis inhibitor 2-DG exacerbated *FoxO1*-deficient macrophages induced higher tumor count. **a** Dynamic B16-F10^mCherry^ counts of co-cultured B16 cells with *FoxO1*^mKO^ (black), control (blue), and *FoxO1*^mKO^ and control pre-treated with 10 mM 2-DG for 1 h (orange and red, respectively) relative to the cell count after 4 h (*n* ≥ 9). **b** HCS images showing the co-culture results of (right) FoxO1^mKO^ macrophages pre-treated with 2-DG and B16-F10^mCherry^ cells (second from the right) *FoxO1*^mKO^ macrophages and B16-F10^mCherry^ cells, (middle) macrophages pre-treated with 2-DG and B16-F10^mCherry^ cells, (second from the left) macrophages and B16-F10^mCherry^ cells, and (left) B16-F10^mCherry^ cells only, at 0, 4, and 28 h. Blue cells are macrophages and red cells are B16-F10 cells. Scale bar, 200 μm. **c** Dynamic B16-F10^mCherry^ counts of co-cultured B16 cells with siRNA-knockout *FoxO1* macrophages (black), control macrophages (blue), and siRNA-knockout *FoxO1* macrophages and control macrophages pre-treated with 10 mM 2-DG for 1 h (orange and red, respectively) relative to the cell count after 4 h (*n* ≥ 9). *P < 0.05; **P < 0.01; ***P < 0.001.

To further confirm the role of *FoxO1* in regulating glucose metabolism in macrophages, we isolated *FoxO1* conditional KO macrophages and detected the metabolic profiles using a Seahorse Glycolysis Stress Test Kit (Agilent). Initially, we measured bioenergetics profiles at the basal state and performed a typical glycolytic function assay^39^ (Fig. 5a). We measured the kinetic extracellular acidification rate (ECAR) response curve in BMDMs from *FoxO1*-deficient mice and littermate controls, and we found that the ECAR was lower in *FoxO1*-deficient macrophages than that in normal macrophages (Fig. 5a). In M2 macrophages, no differences were found (Supplementary Fig. S4F). In *FoxO1*-deficient macrophages, there is a significant decrease in the ECAR of non-glycolytic acidification (Fig. 5b). Moreover, the ECAR by macrophages after the addition of glucose (i.e., due to glycolysis) decreased significantly in *FoxO1*-deficient M0 macrophages (Fig. 5c), and the maximum capacity to generate ATP by glycolysis (glycolytic capacity) also decreased (Fig. 5d). Besides, we found that the oxidative phosphorylation level was higher in *FoxO1*-deficient macrophages than that in normal macrophages, especially the oxygen consumption ratio (Supplementary Fig. S5). In addition, the ATP level of cultured macrophage decreased in *FoxO1*-deficient macrophages (Fig. 5e, f). The link between the metabolic switch and macrophage function was confirmed by experiments with the glycolysis inhibitor 2-Deoxy-D-glucose (2-DG). After pre-treatment with 2-DG, the proportions of B16 cells co-cultured with FoxO1^mKO^ or normal macrophages both increased significantly, which means that the inhibition of glycolysis with 2-DG suppressed the macrophage anti-tumor function including phagocytosis (Fig. 6a, b). In addition, *FoxO1*-deficient macrophages showed a higher B16 cell count than control macrophages, regardless of 2-DG treatment or not (Fig. 6a, b). Although, the inhibited glycolysis activity with 2-DG increased the co-cultured tumor cell counts, which is similar with *FoxO1* deficiency related lower glycolysis level. The *FoxO1*-deficient macrophages still benefited the co-cultured B16 cells compared with 2-DG pre-treated control macrophages, considering the higher OXPHOS activity of *FoxO1*-deficient macrophages (Fig. 6a, b). Furthermore, the same results were obtained for *FoxO1*-small interfering RNA-KO macrophages and control macrophages co-cultured with B16-F10^mCherry^ cells (Fig. 6c).

## Discussion

Our previous research found that *FoxO1* acts as a regulator of anti-tumor effect in TAMs, and we found a connection between hypoxia, *FoxO1*, *MHC-II*, and tumor growth. These results suggested that lower expression of *FoxO1* might be the cause of the pro-tumor M2-like phenotypes of TAMs, but the underlying mechanisms remained unclear^37,38,40,41^. Here we demonstrate that *FoxO1* deficiency causes M0 macrophages to resemble alternatively activated (M2) macrophages through downregulating glycolytic function. We first found a similarity between *FoxO1*^mKO^ M0 macrophages and M2 macrophages at the transcriptome level. As mRNA expression does not always match with protein levels due to the translation process and post-translation modifications^42^, we performed a proteomics analysis to validate our transcriptome results (Fig. 1). The M2-like features of *FoxO1*^mKO^ macrophages, including the inflammatory response, phagocytic activity, and lymphocyte migration, were confirmed by GSEA and quantitative real-time PCR (Fig. 2 and Supplementary Fig. S1). Next, we analyzed migration and phagocytosis using an HCS system. We observed limited migration of *FoxO1*^mKO^ macrophages compared with control macrophages. In addition, phagocytosis was examined in a co-culture system with B16-F10^mCherry^ cells. Although the phagocytosis process could be captured in time-lapse movies (Supplementary Fig. S3C and Supplementary Video S2) and by immunofluorescence results (Supplementary Fig. S3B and Supplementary Video S3A, B), it remained hard to quantify the phagocytosis rate. Hence, we used the relative B16 counts to evaluate the relative phagocytic activity, and we found that *FoxO1*^mKO^ macrophages showed a weaker tumor elimination function (Fig. 3 and Supplementary Video S1A, B). As the metabolic switch functionally shaped the macrophages^11,19^, we next analyzed the GSEA data and found that the glycolytic level was decreased in *FoxO1*^mKO^ macrophages. Combined with our *FoxO1* knockdown results in vitro and in vivo, we verified that *FoxO1* promotes glycolysis in macrophages at transcription, mRNA and protein levels (Figs. 4 and 5, and Supplementary Figs. S4 and S5). Moreover, decreased glycolytic activity of M0 macrophages increased the B16 counts in the co-culture system (Fig. 6).

It has been reported that the metabolic state determines macrophage function, especially glycolysis and oxidative phosphorylation^11,19^. In addition, *FoxO1* was found to be an essential regulator of vascular growth that couples metabolic and proliferative activities in endothelial cells^27^. However, little was known about the regulatory effects of *FoxO1* on the metabolic state of macrophages. Here we discovered that *FoxO1* deficiency significantly decreases the glycolytic activity of macrophages. Using the glycolysis inhibitor 2-DG, we proved that decreased glycolytic activity leads to an increased B16 count in the co-culture system. These results are consistent with previous data of *FoxO1*-KO macrophages, in which *FoxO1* deficiency and relative B16 counts were compared with those of normal resting macrophages (Fig. 6). In summary, our study shows that *FoxO1* regulates anti-tumor effect (including phagocytosis) of macrophages through changing the metabolic hemostasis. This may explain why the upregulation of *FoxO1* is beneficial in several types of cancer^31–33^, i.e., *FoxO1* deficiency drives resting macrophages to an M2-like/TAM-like phenotype and promotes melanoma growth, as proved by our previous work^38^.

Combining the powerful in vitro culture system and multi-omics analysis, we have drawn a connection between *FoxO1*, glycolysis, macrophages and phagocytosis of tumor cells. However, several questions remain. For example, macrophage spontaneous moving speed was measured at three frames per hour, but they moved in random directions, so the speeds we calculated might not reflect their true average speed. Under physiological and pathological conditions, macrophages changed and reached their proper place in a specific environment to perform their function through random migration and directionally migration^43,44^. Macrophage mobility to scavenge nearby bacteria and dead cells is required for effective phagocytosis^45^. Thus, we showed macrophage mobility to partially represent their ability of phagocytosis. However, the relation between *FoxO1*, phagocytosis, random migration, and the chemotactic ability of macrophages remains to be further explored. Then, whether the actual functional readout of *FoxO1* on macrophages function is phagocytosis remains unclear. It’s better to design another definitive and quantitative experiment defining phagocytosis besides the HCS co-culture system of macrophages and B16-F10^mCherry^ cells (Supplementary Fig. S3B, C and Supplementary Videos S2 and S3A, B). Moreover, although we proved reduced glycolysis level and up oxidative phosphorylation level in *FoxO1*-deficient macrophages (Figs. 4 and 5, and Supplementary Figs. S4 and S5), we need more further study on the in vivo effects of *FoxO1* on the metabolic switch and the direct consequences for tumor growth. This could be examined with a TAM-specific glycolysis inhibitor or enhancer paired with *FoxO1* deficiency. In our previous work, we showed that tumor growth accelerated in myeloid *FoxO1* conditional KO mice^38^. Although TAMs exhibited reduced *FoxO1* expression in many tumors, such as mice inoculated with MC38, Hep1-6, or LLC cells (GEO: GSE76033) and human endometrial cancer (GEO: GSE117970), the expression of *FoxO1* in TAMs population remain complicated and heterozygous (Supplementary Fig. S6)^46,47^. These all open up ideas for targeting the metabolism of specific subsets of TAMs through interfering *FoxO1* as potential clinical treatment, such as nanomaterials. Then, the missing of M1 and M2 macrophages in some experiments design led to multiple misunderstanding, it’s better to use those macrophages as control cells in all experiment besides what we had done (Figs. 1 and 3, and Supplementary Fig. S4). Finally, the specific mechanisms by which *FoxO1* deficiency decreases the expression of glycolytic enzymes remain unclear. The fact that we found that the expression of several Wnt genes (*Wnt9a*, *Wnt7a*, and *Wnt5b*) was increased upon *FoxO1* KO, and the observation that Wnt plays an important role in glycolysis^48^ suggests that Wnt signaling is involved, but this hypothesis requires further study. However, *STAT6* and *STAT3* genes showed no difference in our transcriptomics data. Besides, the correlation of *FoxO1* and *STAT6* expression in clustered macrophages from single-cell RNA sequence database of hepatocellular carcinoma and adjacent tissue^49^ showed no difference (Supplementary Fig. S7A). Then, *C/EBPβ* mRNA level decreased in *FoxO1*-deficient macrophages tested by real-time PCR (Supplementary Fig. S7B). *C/EBPβ* is thought as an positive regulator of glycolysis inducing cancer-type metabolic reprogramming^50,51^. Overall, all the above investigations need to be further proved in future research.

According to our experimental results, glycolysis level decreases (Fig. 5), oxidative phosphorylation level increases (Supplementary Fig. S5) and ATP production level (Fig. 5) decreases in *FoxO1*-deficient macrophages. It’s known that the ATP-producing ability of oxidative phosphorylation is much higher than that of glycolysis, the result of ATP reduction is worthy of discussion and analysis. Studies have shown that compared with resting macrophages, the intracellular ATP content of M2 macrophages under *IL-4* polarization is unchanged, but the ratio of AMP/ATP increases and the ratio of ATP/ADP decreases significantly^10^. However, the extracellular ATP content of M1 macrophages under LPS-induced polarization is increased^52^. We can conclude that the actual ATP level of macrophages have nothing to do with the main metabolic pathway. That is, the ATP content of macrophages dominated by glycolysis (relatively low ATP production) is not definitely lower than that dominated by oxidative phosphorylation (relatively high ATP production), because different cells consume different energy in catabolic processes such as signal pathway activation and cytokine secretion. For example, under the condition of *IL-4* stimulation, the macrophage *JAK-STAT6* signaling pathway is activated and the g-phosphate residues of ATP is transferred to *STAT6* for phosphorylation, thereby regulating the downstream signaling pathway^53^.

## Materials and methods

### Mice

C57BL/6 wild-type mice were purchased from Hunan SLAC Laboratory Animal Co., Ltd (China). Lys^Cre/+^ mice (B6.129P2-*Lyz2*^*tm1(cre)Ifo*^/J) were kindly provided by Dr. Bin Gao from NIH, Bethesda, MD. In addition, LoxP-flanked *FoxO1* (FoxO1^fl/fl^) mice were provided by Dr. Qinghua Shi from University of Science and Technology of China (Hefei, Anhui, China). Lys^Cre/+^*FoxO1*^fl/fl^ (*FoxO1*^mKO^ or KO for short) and its littermates Lys^+/+^*FoxO1*^fl/fl^ (control or ctrl) mice used in this study have been previously described^37,38^. Health experimental mice and littermate mice were randomly selected from the mixed feeding cage and used for subsequent research at age of 8–12 weeks. All mice used in this study were bred under specific-pathogen-free conditions according to the guidelines for the Care and Use of Laboratory Animals of the South China University of Technology (Guangzhou, Guangdong, China).

### Cell lines

B16-F10^mCherry^ cell line were kindly provided by Dr. Jun Wang from South China University of Technology. Briefly, B16-F10 cells were infected with mCherry fluorescence expression plasmid packaged lentiviruses and B16-F10^mCherry^ cells were selected with puromycin^54^.

### Bone marrow-derived macrophages

Bone marrow cells were isolated with phosphate-buffered saline (PBS) and red blood cell lysis buffer (Beyotime, China) following a standardized protocol in sterile conditions. In brief, femurs and tibias of hind legs were cut off from killed mice, flushed with PBS, and filtered with 200-mesh filter to get bone marrow cells. Then, cells were centrifuged at 450 × *g* for 5 min, resuspended with 2 mL red blood cell lysis buffer, lysed at 4 °C for 10 min, washed, and resuspended with PBS to eliminate red blood cells. Every 2 × 10^7^ isolated cells were seeded in a 10 cm dish and cultured with L929-conditioned medium (Dulbecco’s modified Eagle medium-high glucose culture medium containing 10% fetal bovine serum, 100 U Penicillin–Streptomycin, and 15% L929 supernatant) for ~7 days, changing culture medium every 2–3 days if necessary. The matured macrophages (M0 macrophage, M0) were digested with 5 mM EDTA at 37 °C for 5 min and then seeded on six-well plates in 1 × 10^6^/ml cell density for stimulation. LPS (100 ng/ml; Peprotech, USA) and 20 ng/ml *IFN-γ* (Peprotech, USA) were used for classic activation macrophage (M1 polarization, M1), whereas 10 ng/ml *IL-4* (Peprotech, USA) and 10 ng/ml *IL-13* (Peprotech, USA) were used for alterative activated macrophage (M2 polarization, M2). The treatment of M0 to polarize towards M1 and M2 with cytokines lasted 24 h. A mixture of BMDMs from two *FoxO1*^mKO^ mice or two littermate mice were used as a sample pool for transcriptomics and proteomics analysis. After the stimulation of BMDMs, RNA was extracted with Trizol reagent (Takara, Japan) and used for microarray analysis with the 4 × 44 K Agilent Whole Mouse Genome Oligo Microarray (SBC, China). Data were obtained through the RPKM (reads per kilobase of transcript, per million mapped read) matching method and collated as Log_2_ values. PCA, heat map, GSEA, and Pearson’s correlation analysis were followed up and analyzed based on the expression of target genes with R. Our transcriptome data can be found on the GEO database (http://www.ncbi.nlm.nih.gov/gds) under the accession number GSE97260.

### Proteomics sample preparation

Cells were gently pelleted with RIPA lysis buffer (Sangon, China) and lysed for 30 min on ice. Lysed cells were centrifuged at 12,000 × *g* for 15 min at 4 °C. The supernatant protein was gently sucked out and quantified with BCA Protein Assay Kit (Thermo, USA). Each aliquot of 50–200 μg of proteins was diluted to 300 μl with UA buffer (8 mol/L urea in 0.1 mol/L Tris-HCl, pH 8.5). The sample solution was centrifuged on a 10k MWCO Concentrator at 10,000 × *g* for 30 min at room temperature, twice. Add 100 μl iodoacetamide (IAA) solution (UA buffer with 50 mM IAA) to the sample solution and allow the resultant solution to stand for 30 min at room temperature in the dark for the alkylation reaction. The solution was removed by centrifugation, then washed twice with 200 μl UA buffer, and wash twice with 300 μl 50 mM NH_4_HCO_3_ buffer at 10,000 × *g* for 30 min at 4 °C. Add 100 μl 50 mM NH_4_HCO_3_ buffer with a trypsin (Thermo, USA)/total protein ratio of ~1 : 50 and incubated at 37 °C overnight for digestion. Then the flow-through fractions by centrifugation at 10,000 × *g* for 15 min were collected and washed with 50 μl of 50 mM NH_4_HCO_3_ buffer twice. Add 10% trifluoroacetic acid (TFA) for a final TFA concentration of 0.4% to stop the digestion. The peptide mixture was captured, concentrated, desalted, and eluted with C18 tips (Thermo, USA), dried with centrifugal-evaporation-concentrator, and stored at −80 °C.

### Liquid chromatography-mass spectrometry analysis

The samples were analyzed using a Q Exactive plus mass spectrometers (Thermo, USA) coupled with an EASY-nLC 1200 HPLC system (Thermo, USA) via a nano-electrospray ion source in data-dependent mode same with previous reports^55,56^. In brief, the concentrated peptide mixture resolved in buffer A (0.1% formic acid (FA)) were loaded onto a 2 cm self-packed trap column (100 μm inner diameter, packed in-house with nanoViper C18, 5 μm) using buffer A and separated on a 15 cm liquid chromatography column (50 μm inner diameter, nanoViper C18, 2 μm) over a 75 min gradient (buffer A, 0.1% FA in water; buffer B, 20% buffer A in Acetonitrile) at a flow rate of 300 nl/min (0–2 min, 2–8% B; 2–47 min, 8–28% B; 47–57 min, 28–44% B; 57–65 min, 44–100% B; and 65–75 min, 100% B). The survey scans were operating with a resolution of 70,000 at 350–1800 m/z and a maximum injection time of 20 ms. Only two to six charge spectra were fragmented with 27% normalized, high-energy collision dissociation in the positive-ion mode. Spectra in the ion trap with an AGC target of 5e5 were acquired for MS^2^ mode and the maximum injection time was 45 ms.

### Proteomics identification by Sequest-HT-based database searching

The mass spectrometry (MS) raw data were searched against the mouse UniProt database (version Jul-03, 2019, 17,019 sequences) using Proteomics Discovery Software (version 2.1, Thermo Fisher Scientific). In the Sequest-HT setting part, we selected trypsin as the proteolytic enzyme and peptides length between 6–144, with 2 missing cleavages sites allowed. The first search mass tolerance and the fragment mass tolerance were 10 p.p.m. and 0.02 Da, respectively. Hereafter, the peptide-spectrum-matches and proteins false discovery rates were set to <1% released and 0.5% strictly.

### Quality control of the MS platform

To confirm the performance of MS, we used the Hela cell lysed peptide mixture (lysed and digested with the same protocol with BMDM samples) as the quality-control standard in each Xcalibur sequence before samples.

### RNA preparation, reverse transcription, and quantitative real-time PCR

BMDMs of *FoxO1*^mKO^ and control mice were lysed and stored with Trizol reagent (Invitrogen, USA) for RNA isolation and subsequent RNA sequence. PrimeScript^RT^ Reagent Kits with gDNA Eraser (Takara, Japan) were used for reverse transcription and quantitative real-time PCR according to the manufacturer’s instructions. Results for all target genes were normalized to that of the housekeeping gene β-Actin was used for calculating ΔΔCt. Samples were excluded from the analysis if Ct value of target genes was out of range. The 5′ to 3′ aligned murine PCR primers (Tsingke, China) used were as follows: *β-Actin*, 5′-TTGCTGACAGGATGCAGAAG-3′ and 5′-ACATCTGCTGGAAGGTGGAC-3′; *FoxO1*, 5′-TGTCAGGCTAAGAGTTAGTGAGCA-3′ and 5′-GGGTGAAGGGCATCTTTG-3′; *LDHA*, 5′-TGTCTCCAGCAAAGACTACTGT-3′ and 5′-GACTGTACTTGACAATGTTGGGA-3′; *PKM2*, 5′-GCCGCCTGGACATTGACTC-3′ and 5′-CCATGAGAGAAATTCAGCCGAG-3′; *Eno1*, 5′-TGCGTCCACTGGCATCTAC-3′ and 5′-CAGAGCAGGCGCAATAGTTTTA-3′; *Glut1*, 5′-CAGTTCGGCTATAACACTGGTG-3′ and 5′-GCCCCCGACAGAGAAGATG-3′; *HK2*, 5′-TGATCGCCTGCTTATTCACGG-3′ and 5′-AACCGCCTAGAAATCTCCAGA-3′; *IL-4*, 5′-GGTCTCAACCCCCAGCTAGT-3′ and 5′-GCCGATGATCTCTCTCAAGTGAT-3′.

### HCS for macrophage tumor-killing function

HCS imaging system (PerkinElmer, USA) was used to analyze the tumor-killing function of KO and control macrophage in confocal mode^57,58^. Every 1 × 10^6^ resuspended polarized macrophages were stained with 2 μl Allophycocyanin (APC)-*F4/80* antibodies (Biolegend, USA; catalog number 123116) for 15 min, at 4 °C after being digested with 5 mM EDTA, 5 min, at 37 °C. Spare antibodies with 1× PBS buffer (PBS), centrifuged at 3000 × *g*, 2 min, were washed. Macrophages on the PerkinElmer 96-well plates with 1 × 10^4^ cells per well in 100 μl culture medium were counted and seeded. Then, 2 × 10^4^ B16-F10-mCherry tumor cells in 100 μl medium were added to the overnight adhered macrophages. The lid was opened and the plate was loaded on the HCS machine immediately, then the image acquisition mode in Harmony software was set. Water objective lens (20×) and R-phycoerythrin (PE) (for cancer cells)/APC (for macrophages)/bright-filed channels were used for confocal imaging mode including 200 ms exposure time, 100% excitation power, and 1.6 μm focus height. Thirteen areas were imaged for each well, with 30 min fixed interval and 96 time points in 37 °C, 5% CO_2_. Cell counts and the tumor cell-killing ability were analyzed with Harmony software according to the cell-tracing analysis model. Displayed view fields were randomly selected from the imaging areas of per well, with 8 h interval after the 8^th^ time point, and the timeline video was composed of representative scene of the wells at 96 time points. All experiments repeated at least twice.

### *FoxO1*-KO virus construction and macrophages infection

*FoxO1*-shRNA plasmids (Sigma, US) were kindly provided by Prof. Ping Gao from SCUT. The used FoxO1-shRNA sequence was 5′-CCGGCGGAGGATTGAACCAGTATAACTCGAGTTATACTGGTTCAATCCTCCGTTTTTG-3′ and 5′-CCGGCCGCCAAACACCAGTCTAAATCTCGAGATTTAGACTGGTGTTTGGCGGTTTTTG-3′. FoxO1-shRNA and NTC (negative control shRNA) plasmids paired with CMV^△R8.91^ (expressing 3 required HIV proteins) and PMD.G (expressing the VSV-G envelop proteins) plasmids were packaged for lentivirus with PEI (polyethyleneimine) in 293T cells. PEI was moved and replaced with normal culture medium 6 h later. Then, lentivirus was isolated from supernatants and cells after 48 and 72 h by 3000 × *g* 5 min centrifugation and filtration with 0.45 μm filter. Six-well plates cultured BMDMs were infected with 1 ml *FoxO1*-shRNA, and NTC lentivirus and 6 μl Turbofect infection reagent (Thermo, US) at the second day to sustain for 24 h, or infected cultured BMDMs with Ad5-CMV-Cre-GFP (Vigenebio, China) or control adeno-virus at 400 multiplicity of infection and sustain overnight. Then the macrophages were cultured in complete culture medium with 15% L929 culture supernatants for another 3 days.

### Glycolysis stress test by seahorse

On the day before acute extracellular flux (XF) assay, seeding 2.0 × 10^5^ cells in growth medium and hydrate sensor cartridge with 200 μl water to incubate overnight in 37 °C without CO_2_. Also, we changed 180 μl/well culture medium to 37 °C pre-warmed bicarbonate-free low-buffered assay medium (pH 7.4) 1 h prior to the XF assay, prepared the compounds and loaded into the hydrated cartridge ports. For Glycolysis Stress Test, final concentrations were 10 mM glucose in port A, 2 μM oligomycin in port B, and 50 mM 2-DG in port C. We created and loaded the assay template on the Seahorse (Agilent, USA), calibrated the sensors with XF calibrant (pH 7.4), and ran the XF assay^39,59^.

### 2-NBDG glucose uptake assay and ATP production assay

2-NBDG (2-(N-(7-Nitrobenz-2-oxa-1,3-diazol-4-yl)Amino)-2-Deoxyglucose), a fluorescent glucose analog, was used to evaluate cell glucose uptake capacity (BioVision, USA). BMDMs were seeded on the 24-well tissue culture plate in 4 × 10^4^/well cell density to tested the glucose uptake level according to the standard operation procedure. The amount of ATP present of viable cells in culture were quantified by CellTiter-Glo® 2.0 Assay (Promega, USA). BMDMs were seeded on the 96-well tissue culture plate in 2 × 10^4^/well or 1 × 10^4^/well cell density to tested the ATP production level according to the standard protocol. ATP solutions (1 μM to 10 nM) were used to generate the standard curve.

### Statistical analysis

All statistical data were presented as mean ± SD. For unpaired samples (*n* ≥ 3), normality test, and homogeneity of variance test were conducted (GraphPad Prism, Version 7.0). If normality and variance homogeneity are met, use two-tailed unpaired Student’s *t*-test; if not, use Mann–Whitney test. For multiple sets of samples, if they meet the normal distribution, use one-way analysis of variance test, otherwise use non-parametric test. *P*-value < 0.05 was considered as statistically significant (*P < 0.05; **P < 0.01; ***P < 0.001).

## Supplementary information

Figure S1

Figure S2

Figure S3

Figure S4

Figure S5

Figure S6

Figure S7

Video S1A

Video S1B

Video S2

Video S3A

Video S3B

## Data Availability

All transcriptomics data were analyzed with R (Version 3.6.3, on the Rstudio platform). After extracting the original data with Proteomics Discovery software, the subsequent graph analysis of the proteome was also completed on R. All code that support the findings of this study are available from the corresponding author upon reasonable request.
