# Supplementary figures and images for: Multi-omics analysis identifies FoxO1 as a regulator of macrophage function through metabolic reprogramming
Source: Cell Death Dis. 2020 Sep 24;11(9):800. doi: 10.1038/s41419-020-02982-0 (PMC7518254; doi:10.1038/s41419-020-02982-0)

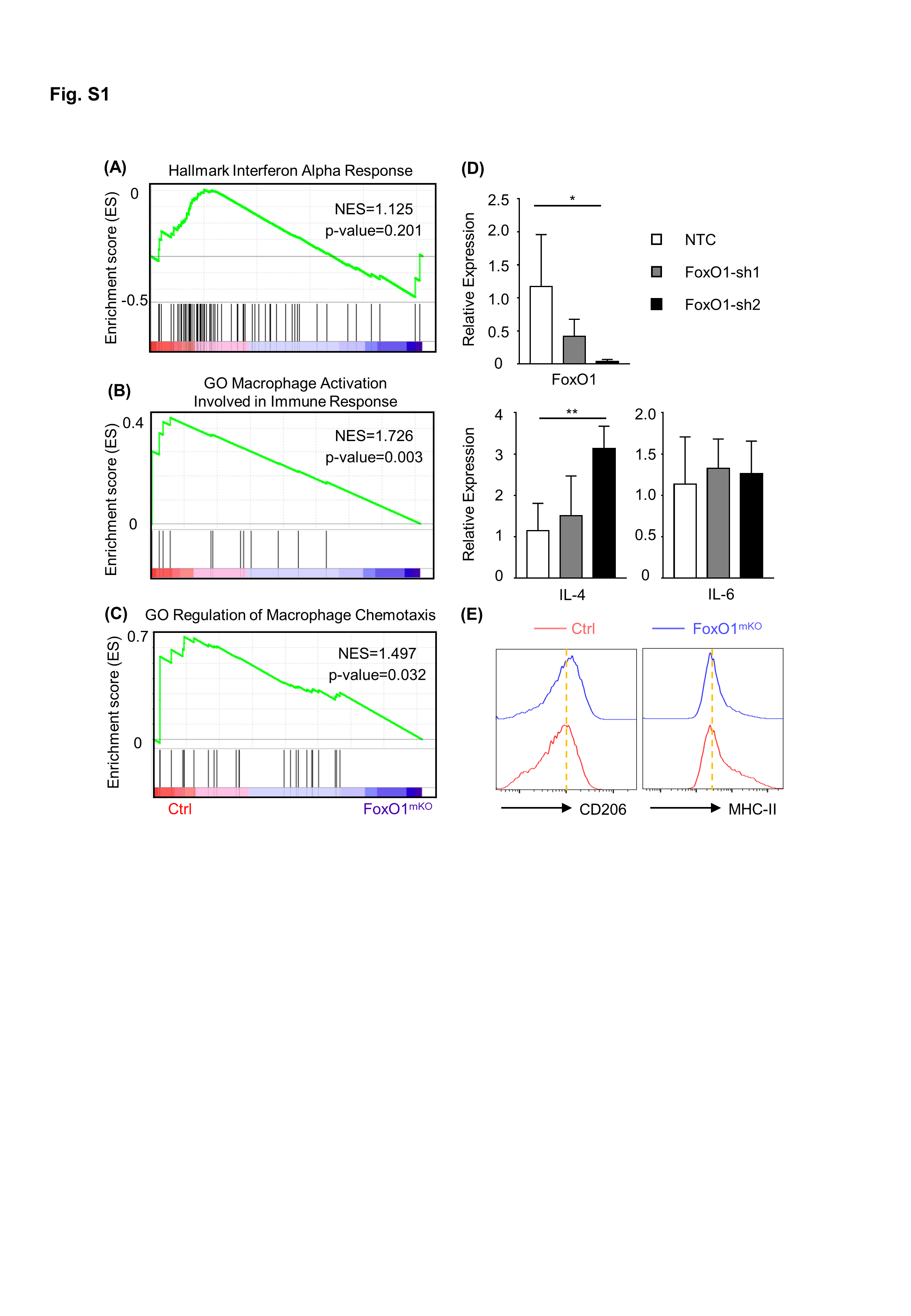

Supplement: Supplementary file 1 — Figure S1 [file 41419_2020_2982_MOESM1_ESM.tif]

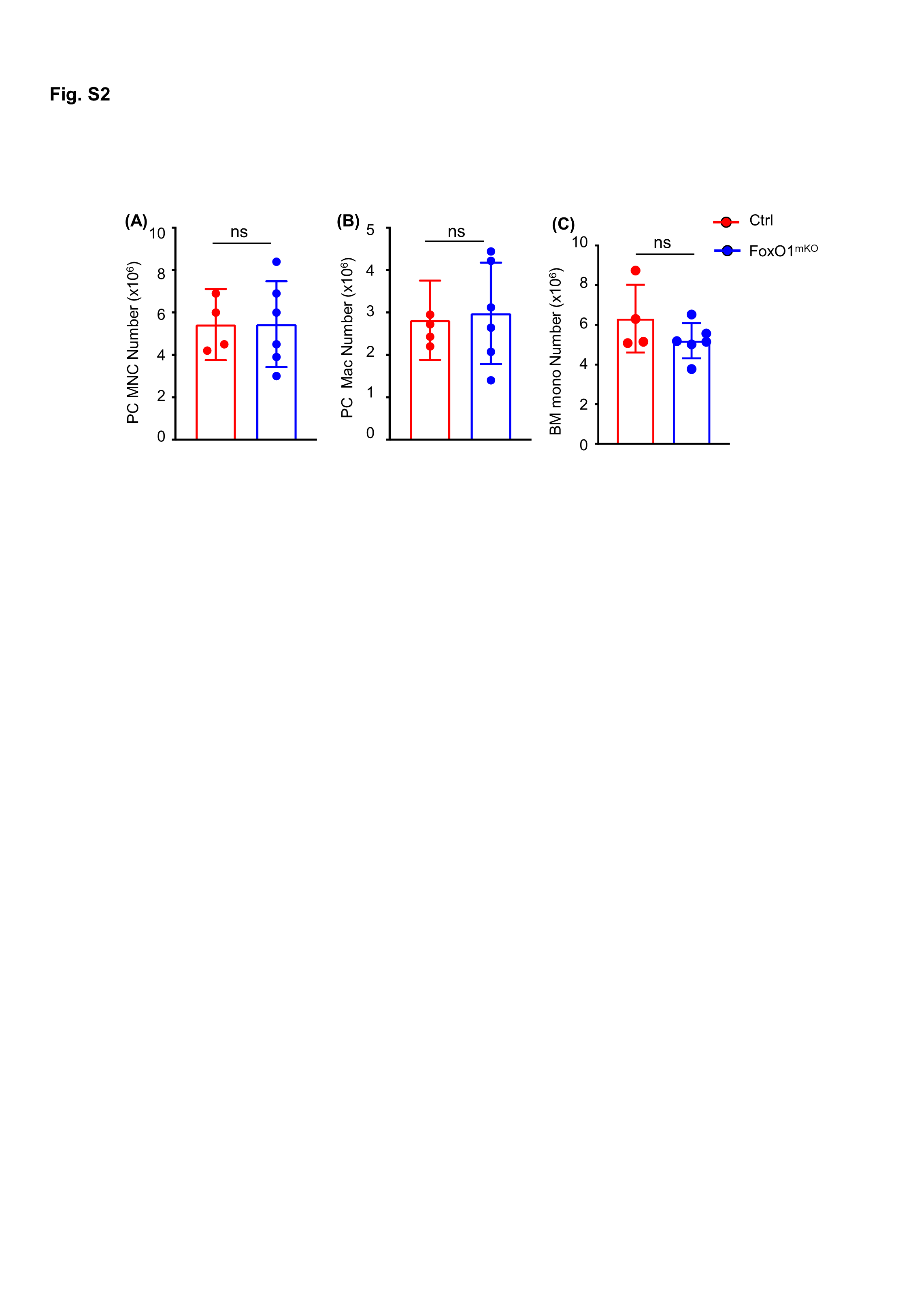

Supplement: Supplementary file 2 — Figure S2 [file 41419_2020_2982_MOESM2_ESM.tif]

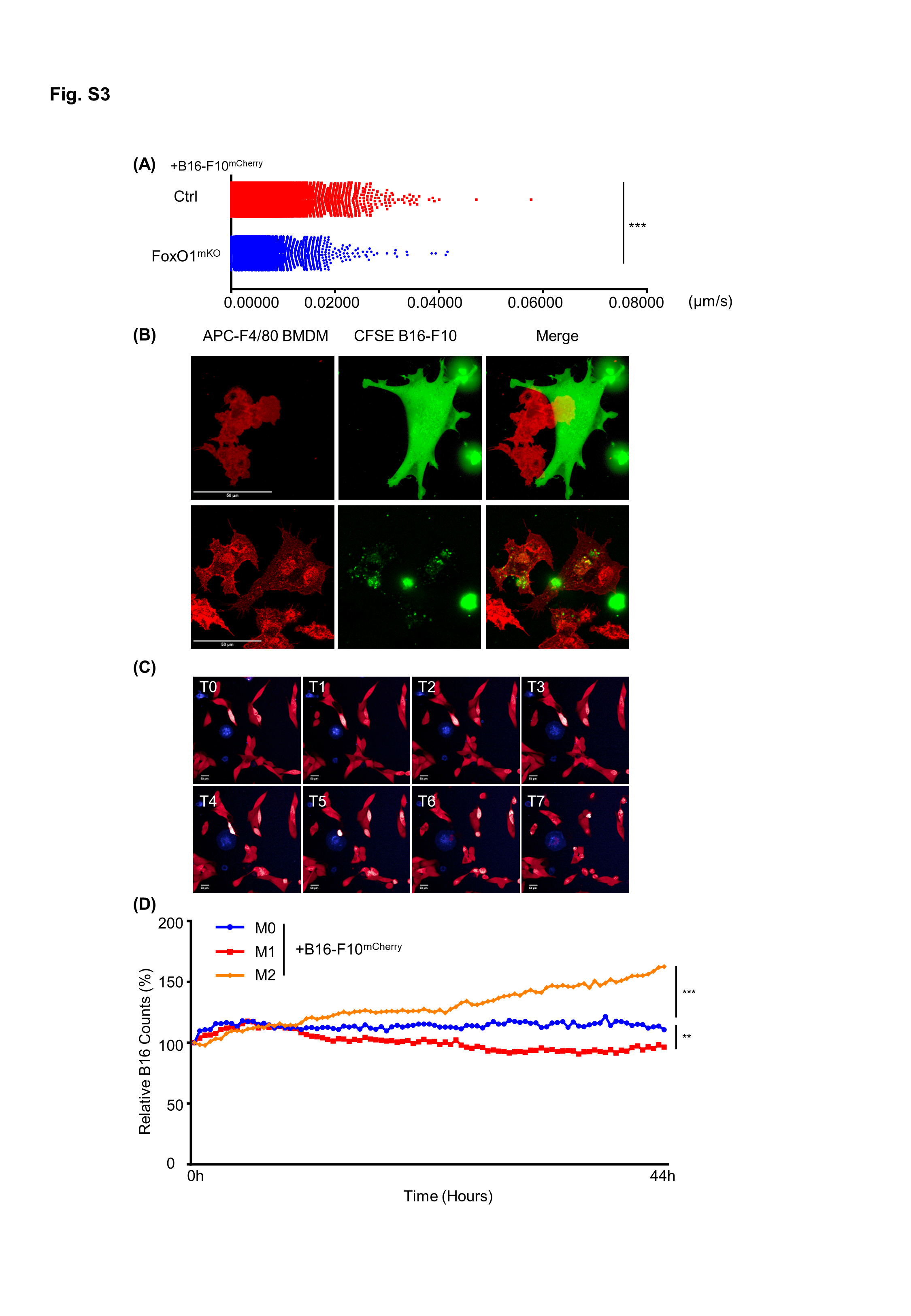

Supplement: Supplementary file 3 — Figure S3 [file 41419_2020_2982_MOESM3_ESM.tif]

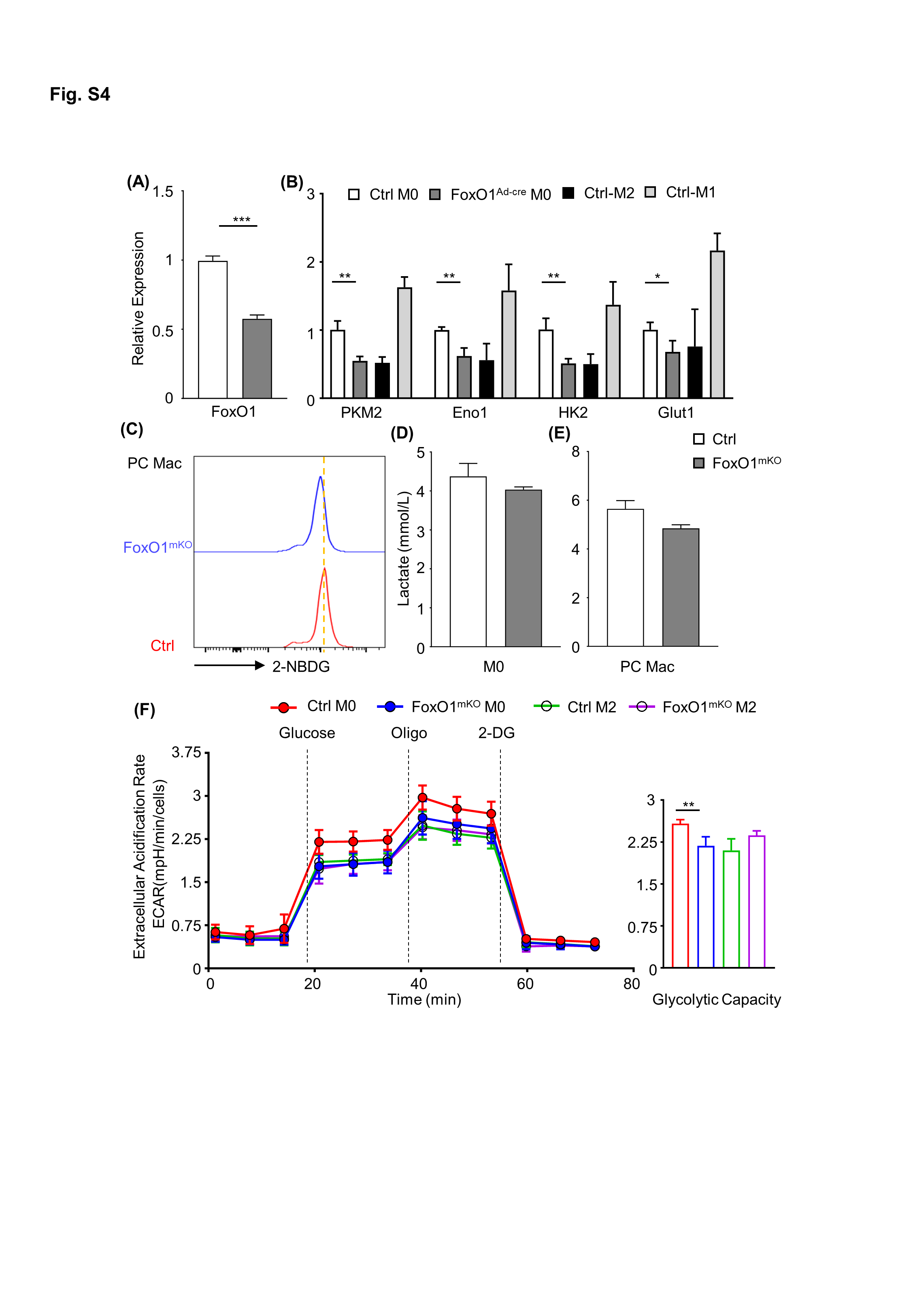

Supplement: Supplementary file 4 — Figure S4 [file 41419_2020_2982_MOESM4_ESM.tif]

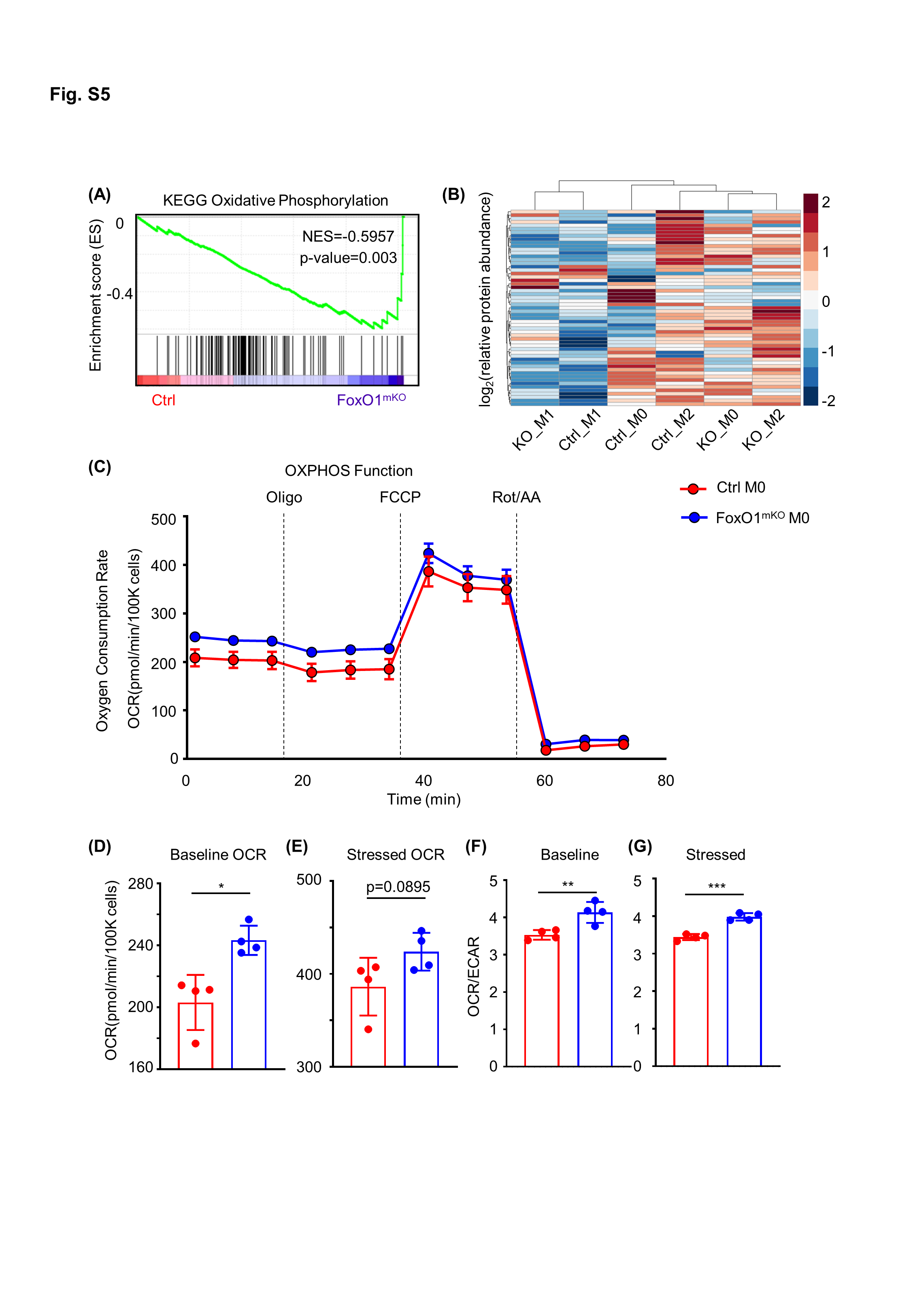

Supplement: Supplementary file 5 — Figure S5 [file 41419_2020_2982_MOESM5_ESM.tif]

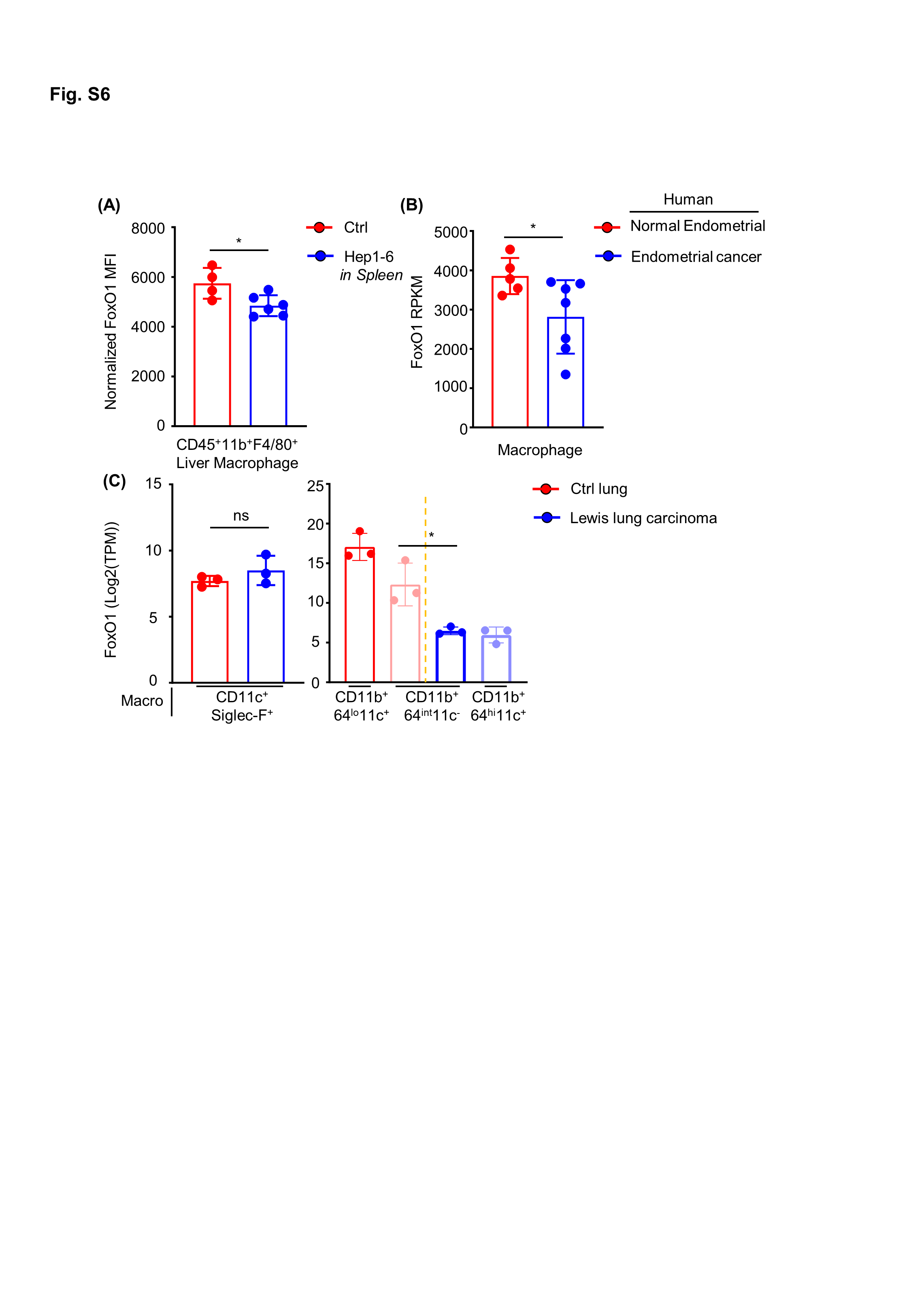

Supplement: Supplementary file 6 — Figure S6 [file 41419_2020_2982_MOESM6_ESM.tif]

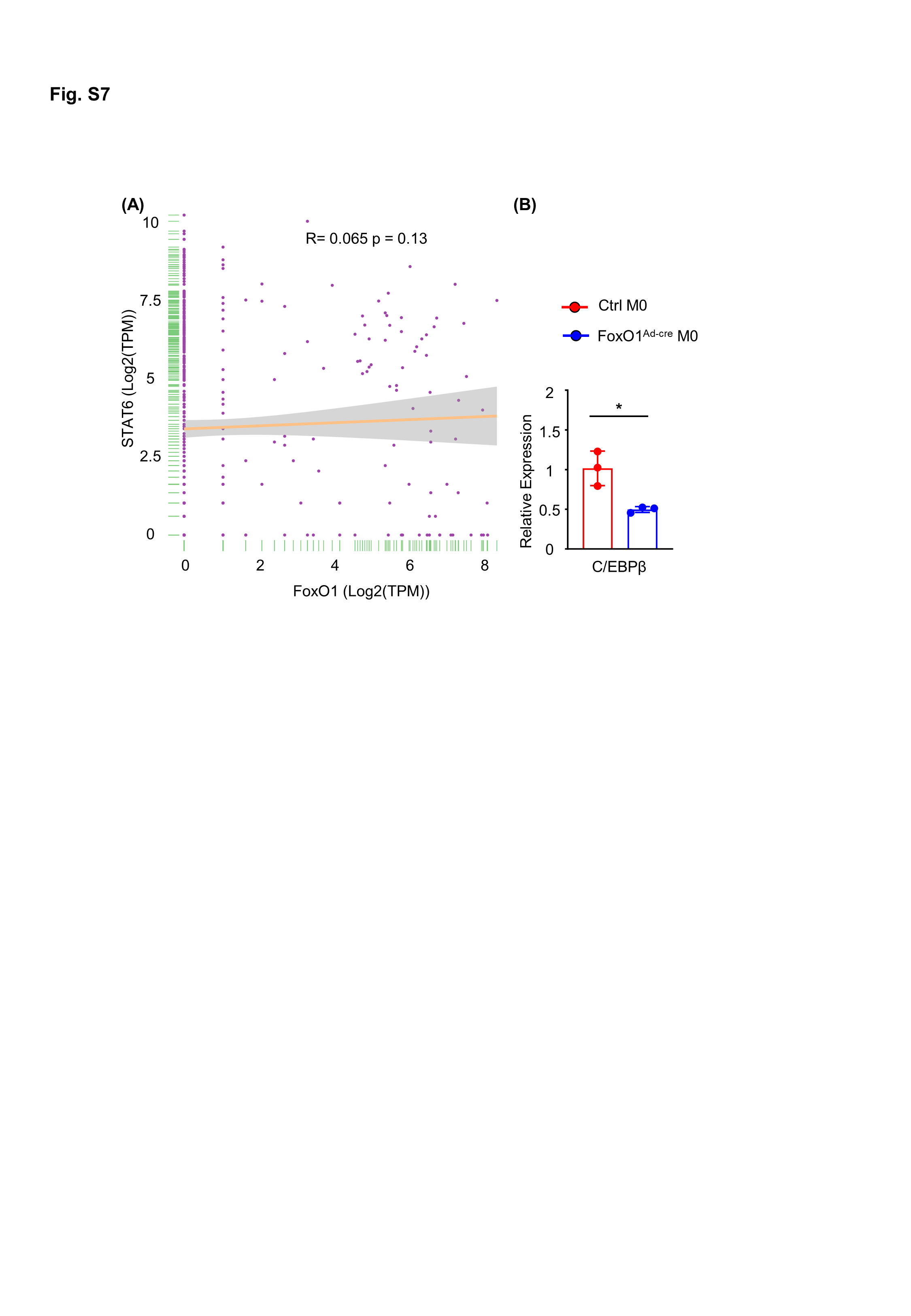

Supplement: Supplementary file 7 — Figure S7 [file 41419_2020_2982_MOESM7_ESM.tif]
